# Trauma and economic displacement: psychological consequences and interventions among Venezuelan refugees in the Americas

**DOI:** 10.3389/fpsyt.2026.1789270

**Published:** 2026-02-16

**Authors:** Gabriel Andrade, Maha Dahawi

**Affiliations:** Ajman University, Ajman, United Arab Emirates

**Keywords:** ecological model, mental health, migration, refugees, stress, Venezuela

## Abstract

This review conceptualizes the Venezuelan refugee crisis as a form of war−like displacement produced by protracted economic collapse, institutional breakdown, and chronic insecurity rather than conventional armed conflict. Synthesizing epidemiological studies and humanitarian reports, it describes consistently elevated rates of depression, anxiety, and trauma−related symptoms among Venezuelan refugees and migrants across the Americas, with prevalences comparable to those observed in conflict−affected refugee populations. The article argues that cumulative exposure to pre−migration deprivation, life−threatening migratory routes such as the Darién Gap, and post−migration legal precarity and discrimination is best understood through ecological and continuous traumatic stress frameworks, which highlight chronic threat and social determinants of mental health. Finally, the review examines emerging evidence on low−intensity, community−based, and culturally adapted interventions, outlining promising scalable approaches as well as structural, systemic, and methodological barriers that currently limit access, effectiveness, and sustainability of care for Venezuelan refugees.

## Introduction

Over the past decade, the Americas have witnessed one of the most significant forced migrations in contemporary history. The large-scale displacement of Venezuelans—driven by a convergence of economic collapse, social instability, and chronic insecurity—has led more than seven million people to leave their country since 2015 (1). Although often framed as an “economic” migration, the Venezuelan exodus shares many psychological features with populations displaced by war or armed conflict. The breakdown of essential services, prolonged exposure to scarcity, and the threat to personal safety generate an enduring sense of collective trauma (2). The phenomenon raises crucial questions about how conventional concepts of war trauma can be adapted to contexts of social collapse and chronic adversity rather than direct warfare. This unprecedented migration outflow, approximating those from war-torn countries like Afghanistan, Ukraine, and Syria, carries profound implications for both the displaced population and the public health infrastructure of receiving countries, especially mental health provision.

In both neighboring and distant host countries, Venezuelan migrants have faced multiple and cumulative stressors across the migration trajectory (3). Many depart after years of witnessing the erosion of community structures and social trust, carrying with them experiences of material deprivation and a loss of identity rooted in dislocation. The migration route itself has become a further source of trauma. Thousands of Venezuelans have crossed the Darién Gap, a dense and perilous jungle corridor between Colombia and Panama that has become a humanitarian flashpoint (4). Reports from humanitarian agencies describe exposure to violence, assault, dehydration, and death along this route (5). Such migratory experiences mirror the life-threatening situations often associated with wartime displacement and illustrate the continuum of trauma that shapes refugees’ psychological vulnerability.

Although the Venezuelan case does not involve classic battlefield operations, its dynamics resonate with what has been described as siege−like conditions in other geopolitical contexts. In Gaza, for instance, a prolonged blockade and recurrent military assaults have produced chronic scarcity of food, medical supplies, and safe shelter, with marked increases in depression, anxiety, post−traumatic stress symptoms, and hopelessness among civilians living under siege (6). Research on Gaza and other besieged settings emphasizes that when populations are trapped in zones of sustained threat and deprivation, resilience is progressively eroded and mental distress becomes widespread and enduring. Similar patterns have been observed in countries subjected to far−reaching sanctions and blockades, such as Iran (7), Cuba (8) and Iraq (9), where restrictions on trade and finances have undermined health systems, limited access to essential medicines, and contributed indirectly to worsening health and psychological outcomes in the general population.

Once in host countries—including Colombia, Peru, Brazil, and increasingly the United States—Venezuelan migrants encounter post-migration stressors that can perpetuate trauma. These include legal uncertainty, employment insecurity, linguistic barriers, and social stigma. Cumulative exposure to pre-migration hardship, migratory trauma, and acculturative stress creates a complex clinical profile that challenges traditional categorizations of posttraumatic stress disorder (PTSD) and adjustment disorders (10). Scholars have proposed that individuals from this crisis experience “continuous traumatic stress,” in which environmental instability sustains hypervigilance and psychological distress (11). Nonetheless, empirical data on the specific psychiatric consequences of Venezuelan displacement remain limited and scattered, particularly compared to other large refugee populations such as Syrians (12) or Rohingya (13).

The present review therefore aims to synthesize current evidence on the mental health impact of the Venezuelan refugee crisis, framing it within models traditionally applied to war and forced displacement. It seeks to maintain an objective and evidence-based perspective by drawing on peer-reviewed data, humanitarian reports, and theoretical frameworks in refugee mental health. Specifically, this work addresses three research questions: 1. What are the predominant patterns and determinants of psychological distress among Venezuelan refugees and migrants across different host contexts?; 2. How can the concept of trauma—originally developed for war-affected populations—be meaningfully applied to individuals displaced by economic and social collapse?; 3. Which psychological and community-based interventions have demonstrated effectiveness or promise in mitigating distress among Venezuelan refugees, and what are the main barriers to their implementation?

## Methods

This narrative review followed a structured, reproducible search strategy to identify empirical and conceptual work on the mental health of Venezuelan refugees and migrants in the Americas. Between September and December 2025, we searched PubMed, PsycINFO, Scopus, and Web of Science using combinations of the following terms in English and Spanish: “Venezuela OR Venezuelan”, “refugee* OR migrant* OR displacement OR diaspora”, “mental health OR depression OR anxiety OR PTSD OR trauma”, and “Latin America OR Americas OR Colombia OR Peru OR Brazil OR Chile OR United States”. We complemented database searches with targeted screening of reference lists of key review articles and grey literature from agencies such as UNHCR, IOM, WHO, and major humanitarian NGOs to capture recent reports and working papers not yet indexed in bibliographic databases. After removal of duplicates, titles and abstracts were screened for relevance, followed by full−text review of potentially eligible papers.

Studies were included if they (1) focused primarily on Venezuelan refugees, migrants, or asylum seekers displaced since 2015; (2) reported quantitative or qualitative data on mental health outcomes, psychosocial distress, or psychological interventions; and (3) were conducted in host or transit countries in the Americas. We excluded editorials without empirical content, purely economic or political analyses without reference to mental health, case reports with fewer than five participants, and studies in which Venezuelans could not be disaggregated from other migrant groups. Data extraction was conducted using a standardized template that captured study design, setting, sample characteristics, measures of mental health, key predictors or contextual factors, and details of any interventions or service models. To enhance reproducibility, the final synthesis organized findings into three domains aligned with the review questions: epidemiology and determinants of distress, conceptualization of trauma, and evidence on interventions and service delivery.

## Results

The database searches yielded approximately 300 records, of which 87 full texts were reviewed and 54 met inclusion criteria for synthesis, alongside relevant reports and policy documents from international organizations. The included literature comprised cross−sectional epidemiological surveys in major host countries, qualitative studies exploring lived experience and idioms of distress, mixed−methods evaluations of psychosocial and community−based interventions, and a smaller number of longitudinal and comparative studies following Venezuelan migrants across different national contexts. Collectively, this corpus documents high and often persistent levels of depression, anxiety, and trauma−related symptoms among Venezuelan refugees, underscores the centrality of cumulative social and structural stressors in shaping mental health outcomes, and provides emerging but uneven evidence for scalable psychological and community−driven interventions within constrained health systems.

## Discussion

### Epidemiology and prevalence of distress

Across host countries, Venezuelan refugees show a marked expansion in both minor and severe mental health symptoms when compared to global and general−population baselines. Population−based estimates suggest that approximately 11–18% of adults worldwide meet criteria for a common mental disorder in a given year, with serious mental illness affecting roughly 4–7% of adults in many countries (14). By contrast, a large meta−analysis of refugees and asylum seekers reported prevalence estimates of about 31% for posttraumatic stress disorder, 31% for depressive disorders, 11–13% for anxiety disorders, and 1.5% for psychosis (15), indicating that clinically significant disorders are several times more frequent in forcibly displaced populations than in the general population. These figures underscore that Venezuelan displacement is unfolding in a broader pattern in which both subthreshold distress and diagnosable psychiatric disorders become highly prevalent under conditions of forced migration and chronic adversity.

Surveys also consistently report elevated symptoms of anxiety, depression, and trauma−related distress among Venezuelan refugees, often several times higher than global population baselines for common mental disorders (16). Border studies at the Ecuador–Peru crossing, for example, have documented anxiety rates around one−third and depression around one−quarter of screened migrants, far above worldwide estimates of 4–5% for each, indicating a syndemic of psychological distress intertwined with material hardship (17).

More recent work with Venezuelan migrants in Peru and Colombia shows high levels of general psychological distress, with some samples reporting that over 90% of participants endorse at least mild symptoms and roughly half meeting thresholds for moderate depression and over half for clinically relevant anxiety (18).

Taken together, these data allow a clearer distinction between the expansion of milder forms of distress and the concentration of more severe or disabling conditions. In Peruvian border samples, for example, around one third of Venezuelan migrants screened positive for generalized anxiety disorder and more than one quarter for clinical depression, compared with global averages of only about 4–5% for each condition (19). Similarly, pooled estimates from refugee meta−analyses indicate that roughly one in three displaced adults meet criteria for major depression or PTSD, whereas lifetime prevalence in community samples is closer to 12% for depressive disorders and 4% for PTSD, with much lower rates of psychosis (15, 20). In the Venezuelan case, the very high proportion of individuals with at least mild symptoms suggests a broad-based elevation of minor distress, while the substantial minority meeting diagnostic thresholds for depression, anxiety, PTSD, or psychosis reflects a concentration of severe mental health problems that exceeds what would be expected in non−displaced populations. These prevalences align with the broader refugee literature, where pooled estimates often report 30–40% rates of depression and PTSD in conflict−affected displaced groups, suggesting that displacement driven by economic collapse and social breakdown can be as psychopathogenic as flight from overt warfare when stressors are cumulative (15).

Comparative studies of Venezuelans in multiple sites (e.g., Colombia vs. United States) indicate that patterns of symptomatology are shaped as much by post−migration context as by pre−migration adversity (21). Perceived discrimination, negative context of reception, and insecure legal status are robust predictors of depressive, anxiety, and posttraumatic symptoms even after controlling for socio−demographic differences such as education. This echoes cross−national refugee data showing that daily stressors—poverty, housing instability, bureaucratic exclusion—often explain more variance in mental health outcomes than the sheer number of potentially traumatic events, underscoring the importance of social determinants (22, 23).

Risk factors repeatedly identified among Venezuelans include irregular migration (e.g., walking for long distances, crossing the Darién or border jungle corridors) (24), female gender (25), pregnancy, informal or exploitative employment, and prior exposure to violence or threats in Venezuela (26). Protective factors, by contrast, include stable documentation, access to employment, social support from co−ethnic networks, and a positive “context of reception,” all of which resonate with ecological models that locate refugee mental health within multilevel systems rather than within individual pathology (27).

### Nature of trauma and stressors

The Venezuelan crisis illustrates how trauma can emerge from protracted societal collapse, chronic insecurity, and institutional fragmentation rather than conventional battlefield exposure. Pre−migration life for many refugees has involved years of escalating scarcity (28), breakdown of health and education services (29), exposure to criminal violence (30), and the erosion of trust in authorities, producing a background of chronic threat and helplessness that blurs the line between “traumatic event” and “daily life.

Post−migration environments in Colombia, Brazil, Peru, Chile, and the United States often remain characterized by legal precarity, underemployment or exploitation, overcrowded housing, and discrimination, which function as chronic, low−grade stressors (31). Ecological and “daily stressors” models argue that such ongoing adversities can maintain or even exacerbate trauma−related symptoms long after initial flight, in some cases overshadowing the impact of discrete pre−migration events (32). This theoretical perspective aligns with “continuous traumatic stress” and related frameworks, which emphasize that for many displaced people, threat is not time−limited but embedded in a persisting social ecology of danger, instability, and uncertainty (33).

Conceptually, the Venezuelan case challenges the event−centric definition of trauma implicit in classic PTSD formulations (34) by foregrounding chronicity, structural violence, and anticipatory anxiety about economic and political futures. It suggests that diagnostic systems and research tools that focus primarily on past, circumscribed events may under−capture suffering arising from prolonged systemic breakdown, thereby underestimating both risk and the need for preventive interventions.

### Cultural dimensions of trauma expression

Latin American idioms of distress such as “*nervios*”, “*tristeza*”, “*desesperanza”*, and “*desahogo*” provide culturally resonant vocabularies through which Venezuelans make sense of suffering associated with displacement and loss (35). These idioms often emphasize embodied symptoms (e.g., somatic tension, gastrointestinal complaints), relational conflicts, and moral/emotional experiences (e.g., feeling “broken” by betrayal or corruption) rather than discrete traumatic flashbacks, highlighting divergence from prototypical Western trauma narratives.

Family, spirituality, and community remain central coping resources within Venezuelan and broader Latin American cultures. Studies of Venezuelan migrants in Peru point to the mobilization of religious faith, the ethos of “pa’lante” (persistence and looking forward), and mutual aid within diaspora networks as important sources of resilience that coexist with high levels of distress (36). Group storytelling, collective rituals, and the practice of *desahogo*—expressing and releasing pent−up emotions—function as communal regulation mechanisms that can either buffer or, in some contexts, amplify distress depending on the presence of validation and safety (37).

These cultural patterns generate significant tensions with Western psychiatric categories that prioritize individually anchored symptoms and discrete diagnoses. Symptom checklists for depression, anxiety, or PTSD may miss context−bound idioms or misclassify adaptive vigilance as pathology when viewed outside the structural conditions that produce threat (38). At the same time, there is a risk of romanticizing idioms and resilience, thereby minimizing real morbidity; the challenge is to integrate local explanatory models with standardized tools in a way that preserves both clinical utility and cultural specificity (39).

A theoretically coherent approach is to view idioms like *nervios* or *desahogo* in the Venezuelan context as culturally shaped surface manifestations of underlying dimensions such as arousal, sadness, or social threat that are partially universal but locally organized. This perspective supports the development of hybrid assessment instruments that embed culturally salient language and examples while retaining links to constructs used in global mental health research, facilitating both culturally competent care and cross−study comparability.

### Evidence on psychological interventions

The intervention literature specifically targeting Venezuelan refugees remains emergent but is growing, with promising results from low−intensity, community−based, and culturally adapted programs in Colombia, Peru, and neighboring countries. A mixed−methods study of Problem Management Plus (PM+), a brief WHO−endorsed intervention delivered by trained lay providers to Venezuelan migrants and Colombian returnees, reported significant reductions in psychological distress and improved functioning, and highlighted the importance of integrating context−specific themes such as migration−related loss, discrimination, and economic insecurity (40).

Within the Venezuelan context, PM+ has typically been delivered in four to five weekly sessions by lay community workers trained and supervised by mental health professionals, using a structured manual that combines problem−solving, behavioral activation, stress−management (e.g., breathing, grounding), and strategies for strengthening social support. In the mixed−methods study with Venezuelan migrants and Colombian returnees, sessions were held in primary care settings and community centers, with homework tasks explicitly linked to migration−related stressors such as navigating documentation procedures, dealing with discrimination in the workplace, or managing overcrowded housing, which participants described as enhancing the perceived relevance of the intervention to their daily lives (40). Group discussions and individual examples from this program suggest that participants valued concrete tools for organizing problems into manageable steps and for “depersonalizing” structural adversities, reporting increased sense of agency despite continued material hardship.

Similarly, the Entre Nosotras intervention offers a concrete example of how group−based psychosocial support can be operationalized for displaced Venezuelan and other Latin American women. The program consists of a series of facilitated small−group sessions that blend psychoeducation about stress, gender−based violence, and rights with collaborative problem−solving around childcare, safety, and livelihood, and incorporates participatory activities such as storytelling, role−plays, and shared rituals to foster trust and mutual aid (41). In the Ecuador and Panama feasibility trial, many participants described the group as a rare ‘protected’ space where they could speak openly about migration−related humiliation and fear without being judged, and qualitative data indicated that perceived improvements in mood and coping were tightly linked to feeling recognized and supported by peers rather than to any single therapeutic technique.

Other initiatives in Colombia and Peru—such as “Entre Nosotras,” “Sin Fronteras,” and “A tu lado”—have combined problem−solving, psychoeducation, and group support to address gender−based violence, parenting stress, and community cohesion among Venezuelan women and families (41). These programs underscore that effectiveness hinges not only on specific therapeutic techniques (often rooted in CBT) but also on community engagement, trust−building, and the incorporation of cultural values around solidarity and spiritual coping (42).

Other NGO−led initiatives tailored to Venezuelan families, such as Sin Fronteras and A tu lado, further illustrate how psychosocial support is embedded in everyday community life. These programs typically combine brief, CBT−informed components—such as identifying unhelpful thoughts, practicing relaxation, or scheduling pleasant activities—with practical guidance on parenting in overcrowded, insecure environments, navigating school and health systems, and connecting families to food, housing, or legal assistance. Group sessions often begin with informal check−ins and shared spiritual or motivational practices (e.g., prayer, collectively invoking the ethos of ‘pa’lante’) and end with concrete action plans for the week, underscoring that what makes these interventions effective is the integration of emotional support with problem−solving around structural stressors and the reinforcement of culturally salient forms of solidarity and hope (42).

While there are as yet few controlled trials of trauma−focused CBT or Narrative Exposure Therapy (NET) specifically in Venezuelan refugee samples, extrapolation from Latinx and refugee populations more broadly suggests that these modalities can reduce PTSD and comorbid symptoms when adapted to language, migration narratives, and family involvement (43). For example, culturally informed trauma−focused CBT with Latinx children adolescents has demonstrated that integrating family values and addressing migration−related stressors enhances therapeutic alliance and symptom improvement (44), offering a template for similar adaptations among Venezuelan youth and adults.

Digital and scalable interventions offer particular promise given the sheer size of the Venezuelan diaspora and the limited availability of specialized providers. WHO’s Self−Help Plus and related guided self−help programs, originally tested in other refugee populations, have shown that brief, low−intensity interventions delivered via groups or digital platforms can yield moderate improvements in distress and functioning, especially when paired with basic needs support (45, 46). Preliminary efforts to deliver remote counseling and psychoeducation to Venezuelan migrants suggest good acceptability but also reveal barriers related to connectivity, privacy in crowded housing, and mistrust of institutions, pointing to the need for careful implementation research.

Methodologically, the current intervention evidence base is constrained by small sample sizes, non−randomized designs, short follow−up, and heterogeneous outcome measures. Few studies stratify results by legal status, gender, or migration route, making it difficult to identify which subgroups benefit most or require tailored approaches. Moreover, many programs are pilot projects run by NGOs or international organizations (47), raising questions about long−term sustainability once external funding ends; integrating successful models into public health and primary care systems remains an unmet challenge.

### Barriers to care and system-level determinants

Structural and administrative barriers substantially limit Venezuelan refugees’ access to mental health care across host countries. Irregular documentation, lack of health insurance, complex registration procedures, and fear of deportation discourage many from seeking formal services, even when distress is severe (18). Linguistic barriers are less pronounced than in other refugee contexts because of shared Spanish in much of Latin America, but differences in vernacular, stigma, and mistrust of authorities still impede help−seeking (48); in the United States, language proficiency and lack of Spanish−speaking clinicians constitute more direct obstacles (49).

Mental health systems in several key host countries—including Colombia, Peru, and Brazil—are underfunded and unevenly distributed (50), with limited specialized services outside major cities and heavy reliance on out−of−pocket payments or fragmented public provision. These constraints intersect with shortages of providers trained in trauma−informed and culturally responsive care for migrants, leading to reliance on emergency or crisis−oriented responses rather than preventive and rehabilitative models.

Stigma and competing survival priorities further depress service utilization. Many Venezuelans frame their suffering in economic or social terms rather than as mental disorders, and may perceive psychological services as a luxury relative to immediate needs like housing or food (51). This is consistent with ecological models emphasizing that without addressing daily stressors and basic rights, demand for formal psychotherapy will remain limited or its effects short−lived (52).

From a systems perspective, integrating trauma−informed, migrant−sensitive mental health care into primary health services, humanitarian assistance, and social protection programs may be more feasible than scaling stand−alone specialist services (53). Task−sharing models that train community health workers, peers, and lay providers to deliver brief interventions (e.g., PM+, basic CBT−informed psychoeducation) offer a pragmatic strategy to expand coverage while maintaining cultural proximity and trust, especially if paired with referral pathways for more complex cases.

### Conceptual integration and future directions

The available evidence also supports a differentiation between the main causes of minor versus severe mental health symptoms among Venezuelan refugees. Subthreshold distress (such as mild anxiety, low mood, and somatic complaints) appears strongly linked to ongoing daily stressors, including poverty, food insecurity, overcrowded housing, and bureaucratic exclusion, which cross−national work has shown to explain more variance in common mental disorders than the sheer number of discrete traumatic events. In contrast, severe conditions such as major depression, PTSD, and psychosis are more closely associated with cumulative exposure to high−impact adversities across the migration trajectory—prolonged deprivation and institutional collapse in Venezuela, life−threatening routes like the Darién Gap, detention or violence in transit, and entrenched discrimination and legal precarity in host countries—mirroring patterns documented in meta−analyses where rates of PTSD and depression reach around 30% and psychosis 1–2% in refugee populations (15). Distinguishing these pathways is crucial for intervention design, as it suggests that population−level strategies targeting social determinants may be particularly effective in reducing widespread minor distress, whereas more intensive, trauma−focused and specialist care is required for the smaller but highly vulnerable group experiencing severe and persistent psychiatric disorders.

In the context of how migration has impacted Venezuelans’ mental health, several key knowledge gaps remain. Longitudinal studies tracking Venezuelan refugees across the migration trajectory and over years in host countries are scarce, limiting understanding of how symptoms evolve, which factors predict chronicity versus recovery, and when interventions are most impactful. Intervention research needs to move beyond pilots to well−powered trials with standardized, culturally validated outcome measures, economic evaluations, and implementation science components that examine scalability, fidelity, and adaptation in real−world systems.

Furthermore, there is a pressing need for cross−cultural psychometric work to adapt and validate instruments for depression, anxiety, PTSD, and functioning in Venezuelan populations, incorporating local idioms and explanatory models. Developing hybrid tools that embed concepts such as *nervios* and *desahogo* while retaining links to global constructs would improve both clinical assessment and research comparability, and help refine theoretical models of continuous traumatic stress in contexts of economic and social collapse.

## Conclusion

The Venezuelan displacement crisis demonstrates that psychological suffering can emerge as powerfully from protracted social collapse as from overt warfare. In addressing the first question laid out at the beginning of this review—what are the predominant patterns and determinants of psychological distress among Venezuelan refugees?—the evidence reveals strikingly high rates of depression, anxiety, and trauma−related symptoms across host contexts, often paralleling those of war−affected populations. These outcomes stem from layered exposures: the erosion of livelihood and safety before migration, the perilous journeys through corridors like the Darién Gap, and the post−migration realities of legal precarity, discrimination, and economic insecurity. Together, these findings point to a complex ecology of cumulative stressors rather than a single cause, emphasizing that chronic threat and social exclusion are key determinants of mental health vulnerability.

The second question asked how the concept of trauma, originally formulated for war−related experiences, can be meaningfully applied to displacement driven by economic and institutional collapse. The Venezuelan case suggests that trauma should be understood not as a discrete event but as an enduring condition of instability and loss. The notion of continuous traumatic stress better captures this lived reality, in which individuals remain exposed to social and structural violence even after migration. This reconceptualization challenges event−centered diagnostic models and expands the terrain of trauma theory to encompass societal breakdown, uncertainty, and moral injury.

Finally, the third question—which interventions show promise and what barriers shape their implementation?—highlights a growing though uneven evidence base. Scalable, community−driven, and culturally adapted interventions such as Problem Management Plus, group psychosocial support, and faith−based initiatives demonstrate feasibility and acceptability, particularly when aligned with values like familismo and collective resilience. Yet constraints in funding, training, and system integration persist. Sustainable progress will require bridging clinical and structural domains, embedding culturally informed care within policies that secure safety, rights, and social inclusion for the Venezuelan diaspora.
